# Primary hyperaldosteronism is associated with increased mortality and morbidity in patients with hypertension and diabetes

**DOI:** 10.3389/fendo.2023.1147225

**Published:** 2023-05-26

**Authors:** Krishnadev Pillai, Ahmed Fares, Soha Dargham, Jassim Al Suwaidi, Amin Jayyousi, Charbel Abi Khalil

**Affiliations:** ^1^ Department of Medicine, Weill Cornell Medicine–Qatar, Doha, Qatar; ^2^ Biostatistics Core, Weill Cornell Medicine–Qatar, Doha, Qatar; ^3^ Heart Hospital, Hamad Medical Corporation, Doha, Qatar; ^4^ Department of Endocrinology, Hamad Medical Corporation, Doha, Qatar; ^5^ Joan and Sanford I. Weill Department of Medicine, Weill Cornell Medicine, New York, NY, United States

**Keywords:** hypertension, cardiovascular disease, diabetes, National Inpatient Database (NIS), primary hyperaldosteronism (PA)

## Abstract

**Aims:**

Primary hyperaldosteronism (PA) is a common cause of hypertension. It is more prevalent in patients with diabetes. We assessed the cardiovascular impact of PA in patients with established hypertension and diabetes.

**Methods:**

Data from the National Inpatient Sample (2008-2016) was used to identify adults with PA with hypertension and diabetes comorbidities and then compared to non-PA patients. The primary outcome was in-hospital death. Secondary outcomes included ischemic stroke, hemorrhagic stroke, acute renal failure, atrial fibrillation, and acute heart failure.

**Results:**

A total of 48,434,503 patients with hypertension and diabetes were included in the analysis, of whom 12,850 (0.03%) were diagnosed with primary hyperaldosteronism (PA). Compared to patients with hypertension and diabetes but no PA, those with PA were more likely to be younger [63(13) vs. 67 (14), male (57.1% vs. 48.3%), and African-Americans (32% vs. 18.5%) (p<0.001 for all). PA was associated with a higher risk of mortality (adjusted OR 1.076 [1.076-1.077]), ischemic stroke [adjusted OR 1.049 (1.049-1.05)], hemorrhagic stroke [adjusted OR 1.05 (1.05-1.051)], acute renal failure [adjusted OR 1.058 (1.058-1.058)], acute heart failure [OR 1.104 (1.104-1.104)], and atrial fibrillation [adjusted OR 1.034 (1.033-1.034)]. As expected, older age and underlying cardiovascular disease were the strongest predictors of mortality. However, the female gender conferred protection [OR 0.889 (0.886-0.892].

**Conclusion:**

Primary hyperaldosteronism in patients with hypertension and diabetes is associated with increased mortality and morbidity.

## 1 Introduction

The autonomous production of aldosterone by an aldosterone-producing adenoma in a single adrenal gland or by bilateral adrenal lesions in hyperaldosteronism. Primary hyperaldosteronism (PA) commonly presents with hypertension and hypokalemia attributed to the effect of aldosterone on the mineralocorticoid receptors resulting in renal reabsorption of sodium and excretion of potassium (1). PA was previously considered a rare cause of hypertension and was estimated to be prevalent in 0.5-2% of hypertensive patients (2, 3). However, with increased use of plasma aldosterone concentration to plasma renin activity ratio or aldosterone/renin ratio as screening tests.

PA is estimated to be present in 5-10% of hypertensive patients (4–8). Hypertension in PA is associated with left ventricular hypertrophy (LVH), stroke, renal dysfunction (9), and impaired glucose metabolism (10). Further, those complications correlate with plasma aldosterone levels. However, there is limited evidence on the co-existence of PA and hypertension in patients with established diabetes. Our study aims to characterize the outcomes of PA in hypertension patients with diabetes using a large database, the National (Nationwide) Inpatient Database (NIS).

## 2 Methods

### 2.1 Data source

We analyzed data from the National Inpatient Sample (NIS) from 2008 to 2016. The NIS is an administrative and de-identified database designed by the Healthcare Cost and Utilization Project (HCUP) to produce US regional and national estimates of inpatient utilization, access, charges, quality, and outcomes, excluding outpatients or readmissions. It accounts for 20% of all US community hospitals. Each entry in the database entails demographic details such as age, gender, race, etiology of admissions, and outcomes while using safeguards to protect the privacy of patients, physicians, and hospitals (11). The study did not require institutional review board approval but an exempt determination (number 18-00017).

### 2.2 Study population and outcomes

We included in our analysis hospitalized patients with both hypertension and diabetes. We then stratified them into two groups according to the presence of PA. The primary outcome was in-hospital death. Secondary outcomes included ischemic stroke, hemorrhagic stroke, acute renal failure, atrial fibrillation, and acute heart failure. Diagnosis, comorbidities, and outcomes were extracted using the ICD-9-CM and ICD-10-CM codes.

### 2.3 Statistical analysis

Data were weighted per the recommendation of the HCUP to generate national estimates of admissions each year (12). Patients were divided into two groups according to the presence of PA and compared in terms of baseline characteristics and outcomes. Finally, we assessed the predictors of mortality in hypertensive patients with diabetes and PA. Data are presented as mean (SD), median (IQR), number (%), or OR (95% CI). A Chi-squared test, student T-test, or ANOVA was used to compare groups, as appropriate. Binary logistic regression was used to calculate the unadjusted odds ratios of outcomes, which were further adjusted for all baseline characteristics that were different among both groups. Multivariate logistic regression was performed to determine predictors of mortality. The significance level was set at 5%; all analyses were done using SPSS version 26 (IBM SPSS Statistics, IBM, Armonk, New York).

## 3 Results

### 3.1 Population

**Figure 1:**
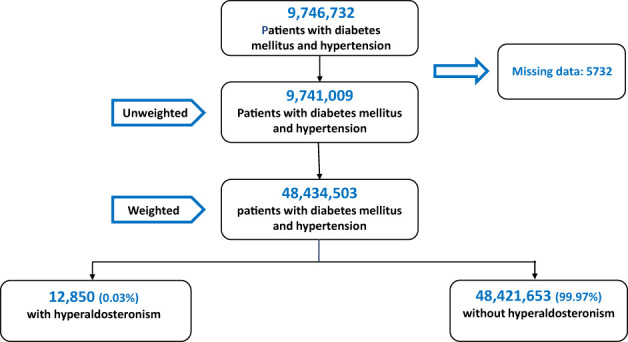
Flow chart of the study.

A total of 9,746,732 patients with diabetes and hypertension were hospitalized between 2008 and 2016. After excluding patients under 18 and those with incomplete or missing records, 9,741,009 patients were included in the analysis. After the application of weights, the data set included 48,434,503 patients. Of these, 12,850 (0.03%) were diagnosed with PA (**Figure 1**).

### 3.2 Baseline characteristics

Patients in the PA group were more likely to be younger [63(13) vs. 67 (14) years], male (57.1% vs. 48.3%), and African-American (32% vs. 18.5%) (p<0.001 for all) (**Table 1**). They were also more likely to have comorbidities such as obesity, dyslipidemia, and renal failure but less likely to smoke or have comorbidities including valvular heart disease, peripheral vascular disease, and coronary artery disease (p < 0.001). As expected, patients with PA had a significantly higher prevalence of hypokalemia than their non-PA counterparts (39.3% vs 9.2%, respectively; p<0.001).

**Table 1:** Comparison of baseline characteristics of patients with diabetes and hypertension, with and without primary hyperaldosteronism (PA).

	With PA	Without PA	P-value
Age			
Mean (SD)	63 (13)	67 (14)	<0.001
<55	3, 322 (25.8%)	8,936,376 (18.5%)	<0.001
55 - 64	3,729 (29.0%)	10,827,343 (22.4%)	<0.001
65 - 74	3,370 (26.2%)	13,073,773 (27.0%)	<0.001
75 - 84	1,974 (15.4%)	10,798,557 (22.3%)	<0.001
>85	457 (3.6%)	4,785,605 (9.9%)	<0.001
Sex			
Male	7,335 (57.1%)	23,367,934 (48.3%)	<0.001
Female	5,515 (42.9%)	25,053,720 (51.7%)	<0.001
Race			
Caucasians	6,343 (54.3%)	28,791,851 (64.6%)	<0.001
African Americans	3,742 (32.0%)	8,232,450 (18.5%)	<0.001
Asians	289 (2.5%)	1074203 (2.4%)	0.114
Hispanics	972 (8.3%)	4,808,929 (10.8%)	<0.001
Comorbidities			
Obesity	3,590 (27.9%)	10,760,842 (22.2%)	<0.001
Dyslipidemia	7,005 (54.5%)	25,360,416 (52.4%)	<0.001
Valvular Heart Disease	633 (4.9%)	2,507,445 (5.2%)	0.007
Peripheral Vascular Disease	1,107 (8.6%)	5,249,702 (10.8%)	<0.001
Chronic kidney disease	5,274 (41.0%)	13,559,885 (28.0%)	<0.001
Coronary Artery Disease	2,830 (22.0%)	13,661,526 (28.2%)	<0.001
Aortic aneurysm	139 (1.1%)	494,591 (1.0%)	0.497
Hypokalemia	5,046 (39.3%)	4,476,475 (9.2%)	<0.001

### 3.3 Comparison of PA and non-PA Groups

Patients with PA were more likely to die in hospital [adjusted OR = 1.076 (1.076-1.077)] and experience adverse outcomes, including atrial fibrillation [adjusted OR = 1.034 (1.033-1.034)], ischemic stroke [adjusted OR = 1.049 (1.049-1.05)], hemorrhagic stroke [adjusted OR = 1.05 (1.05-1.051)], acute renal failure [adjusted OR = 1.058 (1.058-1.058)], and acute heart failure [adjusted OR = 1.104 (1.104-1.104) (**Table 2**).

**Table 2:** Comparison of outcomes of patients with diabetes and hypertension with and without primary aldosteronism (PA).

	Number of Events (OR; 95% CI)			
Outcome	With PA	Without PA	Adjusted OR (95% CI)	P-value
**Mortality**	1,211,127	125	1.076 (1.076-1.077)	<0.001
	(OR = 1)	(OR = 0.38; 0.256-0.564)		
**Atrial Fibrillation**	7,853,993	1,902	1.034 (1.033-1.034)	<0.001
	(OR = 1)	(OR = 0.892; 0.800-0.994)		
**Ischemic Stroke**	278,901	89	1.049 (1.049-1.05)	<0.001
	(OR = 1)	(OR = 1.208; 0.760-1.921)		
**Hemorrhagic Stroke**	87,865	54	1.05 (1.05-1.051)	<0.001
	(OR = 1)	(OR = 2.349; 1.299-4.248)		
**Acute renal failure**	7494,970	2,863	1.058 (1.058-1.058)	<0.001
	(OR = 1)	(OR = 1.563; 1.425-1.715)		
**Acute Heart Failure**	12,024,478	3,108	1.104 (1.104-1.104)	<0.001
	(OR = 1)	(OR = 0.962; 0.879-1.052)		

### 3.4 Predictors of mortality

As expected, the mortality risk in patients with PA increases with age [OR = 1.039 (1.027-1.046)] (**Table 3**). Compared to Caucasians, Asians have a higher mortality risk [OR = 1.375 (1.361-1.389)]. Interestingly, African American and Hispanic patients had a lower risk of mortality [ORs = 0.874 (0.869-0.878) and 0.93 (0.924-0.936), respectively]. The female gender conferred protection [OR 0.889 (0.886-0.892]. Among comorbidities, valvular heart disease, peripheral vascular disease, chronic kidney disease, coronary artery disease, and hypokalemia were associated with increased mortality. Interestingly, obesity and dyslipidemia were shown to be protective.

**Table 3:** Predictors of mortality in patients with diabetes, hypertension, and primary aldosteronism.

		OR (95% CI)	P-value
**Age**	*Mean*	1.039 (1.027-1.046)	<0.001
	*<55*	Ref	Ref
	*55 - 64*	1.631 (1.619-1.644)	<0.001
	*65 - 74*	2.231 (2.214-2.247)	<0.001
	*75 - 84*	3.221 (3.198-3.244)	<0.001
	*>85*	4.872 (4.836-4.909)	<0.001
**Sex**	Male	Ref	Ref
	Female	0.889 (0.886-0.892)	<0.001
**Race**	Caucasian	Ref	Ref
	African American	0.874 (0.869-0.878)	<0.001
	Hispanic	0.93 (0.924-0.936)	<0.001
	Asian	1.375 (1.361-1.389)	<0.001
**Obesity**	No	Ref	Ref
	Yes	0.645 (0.641-0.648)	<0.001
**Dyslipidemia**	No	Ref	Ref
	Yes	0.617 (0.615-0.619)	<0.001
**Valvular Heart Disease**	No	Ref	Ref
	Yes	1.658 (1.647-1.669)	<0.001
**Peripheral Vascular Disease**	No	Ref	Ref
	Yes	1.474 (1.467-1.482)	<0.001
**Chronic kidney disease**	No	Ref	Ref
	Yes	2.004 (1.996-2.011)	<0.001
**Coronary Artery Disease**	No	Ref	Ref
	Yes	1.218 (1.214-1.223)	<0.001
**Hypokalemia**	No	Ref	Ref
	Yes	1.20 (1.01-1.31)	<0.001

## 4 Discussion

Our study showed that PA among hospitalized patients with hypertension and diabetes was associated with increased mortality risk, atrial fibrillation, stroke, acute heart failure, and acute renal failure. Comorbidities, including valvular heart disease, peripheral vascular disease, chronic kidney disease, and hypokalemia, independently increased mortality risk. It is already known that hypertension in the presence of PA is associated with higher cardiovascular event rates. Milliez et al. reported an increased risk of stroke, myocardial infarction, and atrial fibrillation in patients with PA compared to patients with essential hypertension (13).

HA is more frequent in females than in females. However, we observed a greater prevalence of the disease in males. One plausible explanation for this discordance is that we only assessed in-hospital patients with diabetes and PA; therefore, the male/female ratio could differ from the one observed in cohorts from outpatient clinics. Further, data pertinent to the gender difference in primary HA with diabetes are lacking. A recent Taiwanese analysis estimated the male proportion of diabetes patients with PA to be 50.8% (14). In an analysis of 256 outpatients with new-onset T2D and hypertension patients, a higher number of males with PA was reported (15).

In our study, PA patients had a higher rate of hypokalemia, increasing their mortality risk. This finding was recently reported in a 10-year follow-up of a cohort of PA patients (16). However, we did not find any difference in the prevalence of aortal aneurysms. Previous data indicated that PA patients had larger ascending aorta diameters and increased abdominal aortic calcification (17, 18), facts that, unfortunately, we can’t check in the NIS database since those data are lacking. In experimental models, excessive aldosterone levels were related to myocardial and vascular inflammation, injury, and fibrosis (19–21). Aldosterone-mediated fibrosis of the human myocardium has also been reported in cardiac imaging studies and is associated with systolic and diastolic dysfunction (22, 23). The structural changes of the heart in the setting of PA serve as a substrate for cardiac arrhythmias such as atrial fibrillation. Hypokalemia, one of the hallmarks of PA, causes prolongation of the atrioventricular conduction time and increases the likelihood of atrioventricular reentry mechanisms, increasing the risk of atrial fibrillation (24). Furthermore, aldosterone directly contributes to cardiac electrophysiologic remodeling, increasing the risk of atrial fibrillation (25). Aldosterone also induces inflammation, fibrosis, and necrosis of various other organs, including the kidneys (26), which may explain the increased risk of acute renal failure.

The pathophysiology of diabetes in PA is multifactorial. In 1964, Conn et al. reported an increased incidence of impaired glucose tolerance in a review of 145 patients with PA (1). The higher prevalence of diabetes in PA can be explained by impaired pancreatic insulin secretion and reduced insulin sensitivity in the setting of high levels of aldosterone (27). Garg et al. demonstrated that elevated aldosterone levels were associated with insulin resistance in normotensive healthy subjects (28). In a 10-year prospective study, high plasma aldosterone levels predicted the development of insulin resistance, suggesting that hyperaldosteronism may induce diabetes (29). Aldosterone also induces clonal β-cell failure through the glucocorticoid receptor (30).

The exact mechanisms by which PA increases cardiovascular mortality and morbidity are unclear. It might be possible that PA-related inflammation mediated by higher aldosterone levels accelerates atherosclerosis in diabetes since aldosterone promotes the production of inflammatory cytokines and decreases beneficial adipokines such as adiponectin (31). Further, high aldosterone levels are associated with increased reactive oxygen species (32–34), one of the hallmarks of the development of cardiovascular complications of diabetes.

The results of our study must be interpreted within its limitations. We used ICD-9-CM and ICD-10-CM codes to extract data from the NIS database; reporting and coding errors may be present. Further, the methodology and criteria used to diagnose PA are unclear. The retrospective nature of the database prevents us from establishing causality, and only correlations can be made. Due to the unavailability of patient-level data, information related to medications, biochemical parameters, echocardiography data, diabetes control and duration, and severity of comorbidities were not included. Our analysis only included hospitalized patients, which may represent a sicker cohort with an increased prevalence of adverse outcomes compared to the general population. Lastly, data following patient discharge or readmission data were unavailable, so follow-up of patients was not possible. Despite these limitations, our results are derived from a large sample representative of the US population.

## 5 Conclusion

In conclusion, we showed that primary hyperaldosteronism is associated with worse outcomes in hospitalized patients with hypertension and diabetes.

## Data availability statement

The original contributions presented in the study are included in the article/supplementary material. Further inquiries can be directed to the corresponding author.

## Ethics statement

The study did not require institutional review board approval but an exempt determination (number 18-00017). Written informed consent for participation was not required for this study in accordance with the national legislation and the institutional requirements.

## Author contributions

CAK conceived the study concept and design. KP and AF acquired data and performed statistical analyses with SD. KP, AF, JA, AJ, and CAK analyzed and interpreted data. KP and AF wrote the first draft and conducted the literature search. CAK is the guarantor of this work and, as such, has full access to all the data in the study and takes responsibility for the integrity of the data and the accuracy of the data analysis. All authors contributed to the article and approved the submitted version.
